# Acquired partial lipoatrophy as graft-versus-host disease and treatment with metreleptin: two case reports

**DOI:** 10.1186/s13256-018-1901-y

**Published:** 2018-12-14

**Authors:** Yusuke Shibata, Atsuko Nakatsuka, Jun Eguchi, Satoshi Miyamoto, Yukari Masuda, Motoharu Awazawa, Akinobu Takaki, Ryuichi Yoshida, Takahito Yagi, Jun Wada

**Affiliations:** 1 Department of Nephrology, Rheumatology, Endocrinology and Metabolism, Okayama University Graduate School of Medicine, Dentistry and Pharmaceutical Sciences, 2-5-1 Shikata-cho, Kita-ku, Okayama, 700-8558 Japan; 2 Department of Metabolic Diseases, Graduate School of Medicine, The University of Tokyo, Tokyo, 113-8655 Japan; 3 Department of Gastroenterology and Hepatology, Okayama University Graduate School of Medicine, Dentistry, and Pharmaceutical Sciences, Okayama, 700-8558 Japan; 4 Department of Gastroenterological Surgery, Okayama University Graduate School of Medicine, Dentistry and Pharmaceutical Sciences, Okayama, 700-8558 Japan

**Keywords:** Lipoatrophy, Diabetes, Transplantation, Leptin, Insulin resistance

## Abstract

**Introduction:**

Acquired partial lipoatrophy has been reported after bone marrow transplantation during childhood; however, no adult cases have previously been reported. We herein report two adult cases of acquired partial lipoatrophy after transplantation.

**Case presentation:**

A 28-year-old Japanese woman developed diabetic ketoacidosis and received insulin therapy after bone marrow transplantation. She manifested partial lipoatrophy of the extremities, prominent insulin resistance, hyperglycemia, hypertriglyceridemia, and fatty liver. A 40-year-old Japanese woman underwent liver transplantation from a living donor for alcoholic liver disease after abstinence from alcohol. She newly developed non-alcoholic steatohepatitis and diabetes. Non-alcoholic steatohepatitis progressed to liver failure, and a second liver transplantation from a brain-dead donor was performed at 42 years of age. She demonstrated loss of subdermal fat of the upper and lower extremities, prominent insulin resistance, hyperglycemia, and hypertriglyceridemia. In both cases, the injection of recombinant methionyl human leptin reversed all of the metabolic abnormalities.

**Conclusions:**

Acquired partial lipoatrophy after transplantation is a manifestation of chronic graft-versus-host disease in adults. This entity is associated with diabetes with prominent insulin resistance and severe hypertriglycemia and can be successfully treated with metreleptin for the long term.

## Introduction

Lipodystrophic disorders are characterized by partial or total lipoatrophy, diabetes with prominent insulin resistance, acanthosis nigricans, hypertriglyceridemia, cardiomegaly, and severe fatty liver. Lipodystrophic disorders are further classified into two main categories: familial/congenital and acquired lipoatrophy. Although patients with lipoatrophy do not tend to be obese, they are associated with obesity-related metabolic abnormalities, such as insulin resistance, hyperglycemia, and hypertriglyceridemia. Congenital lipodystrophic disorders are rare, with acquired lipoatrophy caused by human immunodeficiency virus (HIV) and the use of highly active antiretroviral therapy (HAART) being more common, referred to as HIV-associated lipoatrophy [1]. In addition to HIV, patients with autoimmune diseases also develop a type of total lipoatrophy called Lawrence syndrome [2]. This suggests that abnormalities in the immune system are closely linked to the development of acquired lipoatrophy.

There have been some case reports and case series of patients who developed partial lipoatrophy after hematopoietic stem cell transplantation during childhood [3–6]. Acquired partial lipoatrophy has been reported to be associated with symptoms of chronic graft-versus-host disease (GVHD) [3], suggesting that acquired partial lipoatrophy after bone marrow or organ transplantation may be a manifestation of GVHD.

We herein report two adult cases of acquired partial lipoatrophy—one after bone marrow and one after liver transplantation. They were successfully treated with the injection of recombinant methionyl human leptin.

## Case presentation

### Case 1

A 28-year-old Japanese woman was referred to our hospital for the treatment of hyperglycemia and partial lipoatrophy. She had acute promyelocytic leukemia and had received allogeneic bone marrow transplantation from her older brother at 4 years of age. When she was 19 years of age, she developed diabetic ketoacidosis and started insulin injection therapy. Although she had been treated with daily doses of 40 units of insulin detemir, 30 units of insulin lispro, 50 mg of sitagliptin, 15 mg of pioglitazone, 750 mg of metformin, and 200 mg of bezafibrate, her hemoglobin A1c and serum triglyceride levels remained high, ranging between 8.5 and 9.0% and 900 and 1000 mg/dL, respectively. Under nutritional guidance, she had been on a 1600 kcal diet consisting of 60% carbohydrates, 20% protein, and 20% fat.

**Fig. 1:**
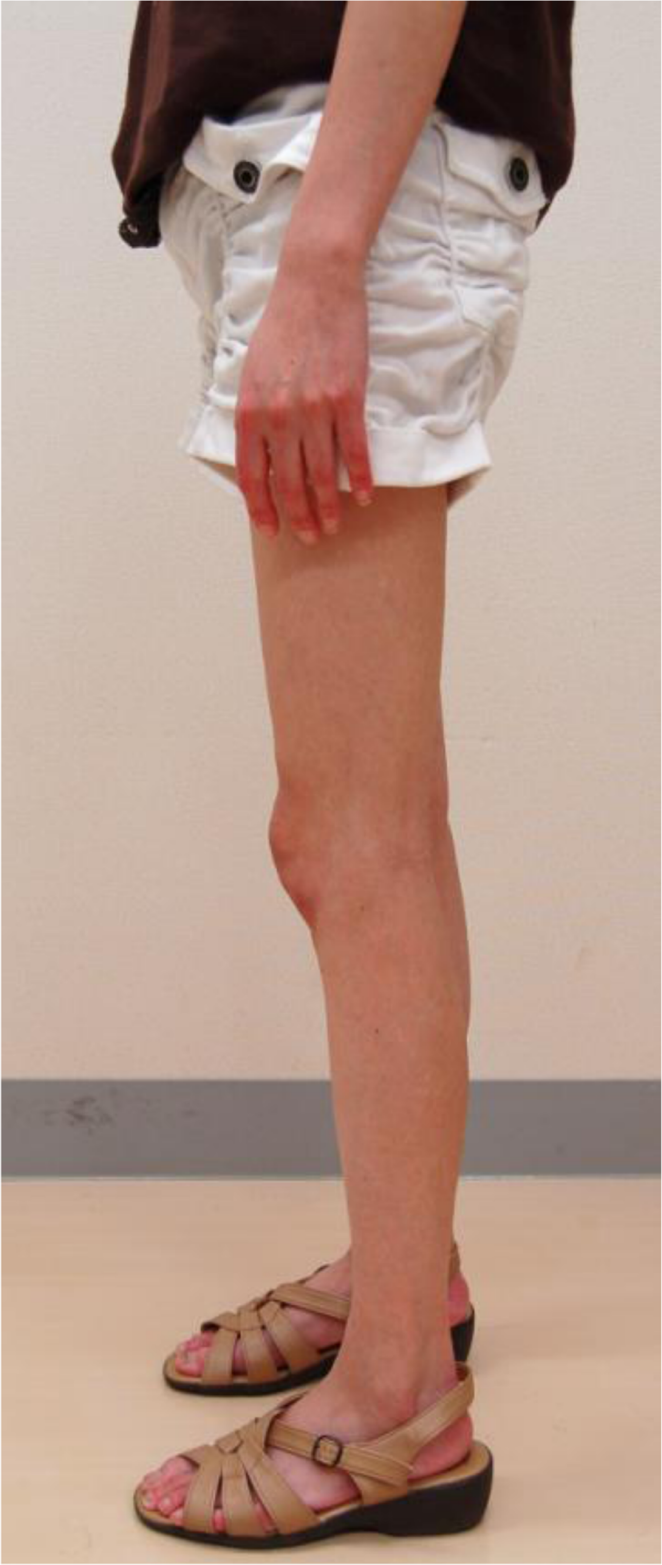
Loss of subdermal adipose tissues in the bilateral forearms and lower legs in Case 1. The upper arms, thighs, face, and trunk were spared from lipoatrophy

On admission, her height was 158 cm, body weight 42.6 kg, body mass index (BMI) 16.9 kg/m^2^, and systemic blood pressure 122/75 mmHg. She manifested almost complete loss of subdermal adipose tissues of the bilateral forearms and lower legs, but her upper arms, thighs, face, and trunk were spared from lipoatrophy (Fig. 1). Her fasting glucose was 232 mg/dL and HbA1c 8.7%. Serum C-reactive protein (CRP) was 2.8 ng/mL, ΔCRP 2.7 ng/mL after the injection of glucagon, and daily urinary excretion 80.9 μg/day, while her serum leptin levels was 6.5 ng/mL (range for women, 2.5–21.8). She had no elevation of liver enzymes: aspartate transaminase (AST) 21 IU/L, alanine aminotransferase (ALT) 19 IU/L, and gamma-glutamyl transferase (GGT) 32 IU/L.

**Fig. 2:**
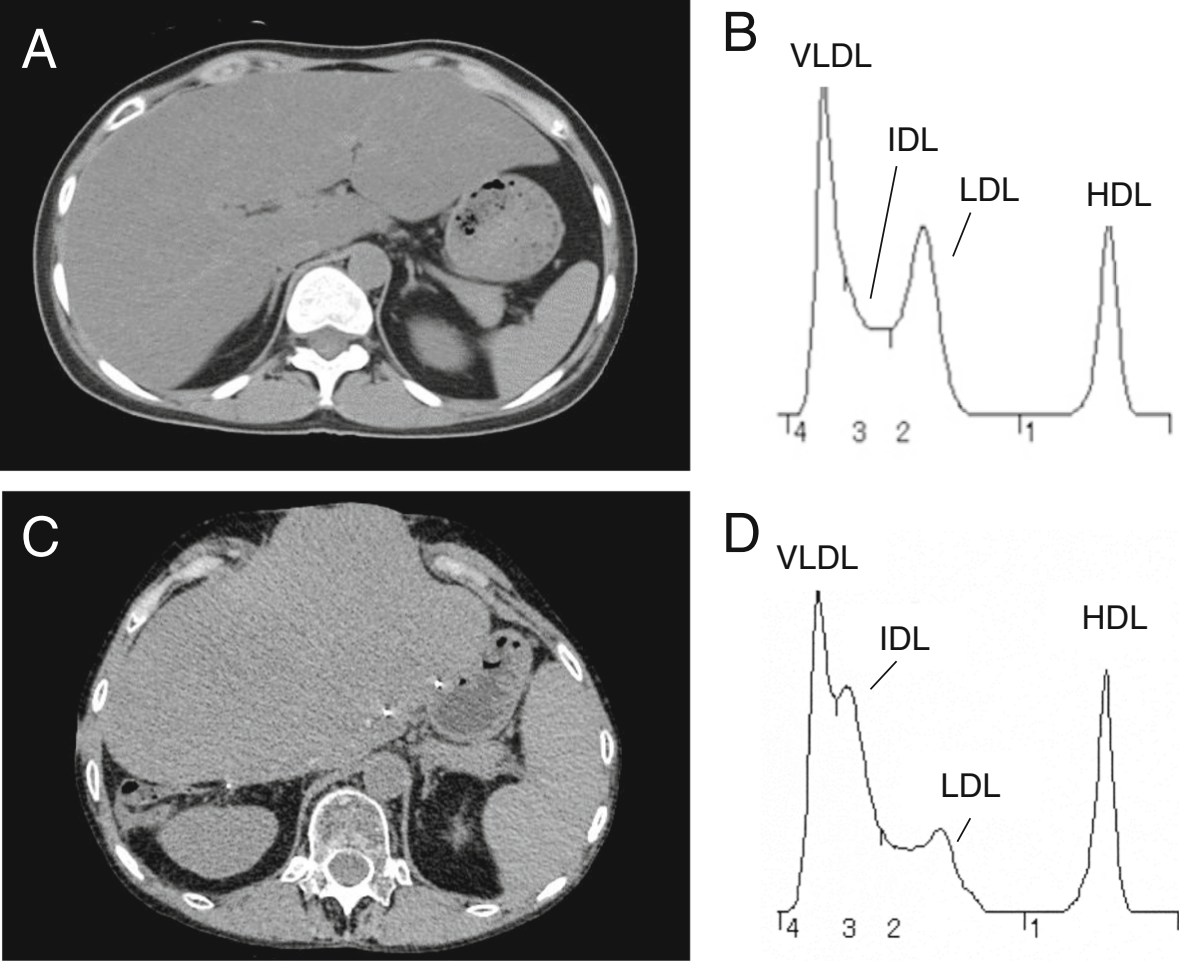
Computed tomography and polyacrylamide gel electrophoresis of two cases with lipoatrophy. **a** and **c**. Abdominal computed tomography of Case 1 (**a**) and Case 2 (**c**). Prominent fatty liver is observed. **b** and **d**. Acrylamide gel electrophoresis of Case 1 (**b**) and Case 2 (**d**). The elevation of VLDL and IDL is notable. *IDL* intermediate-density lipoprotein, *LDL* low-density lipoprotein, *VLDL* very low-density lipoprotein

However, reduced subdermal adipose tissues (47.3 cm^2^) and the accumulation of visceral adipose tissues (99.3 cm^2^) along with notable fatty liver were found by computed tomography (Fig. 2a). She also had dyslipidemia with low high-density lipoprotein (HDL) cholesterol (40 mg/dL) and high triglyceride (968 mg/dL) levels. Acrylamide gel electrophoresis demonstrated the elevation of very low-density lipoprotein (VLDL) and intermediate-density lipoprotein (IDL) (Fig. 2b). A euglycemic hyperinsulinemic clamp study targeting a plasma glucose concentration of 100 mg/dL with an insulin infusion rate of 1.25 mU/kg/min suggested a prominent insulin resistance, since a glucose infusion rate of 2.32 mg/kg/min (normal range 8.0–12.0 mg/kg/min) was required to maintain the target glucose levels. The presence of hyperglycemia, prominent insulin resistance, hypertriglyceridemia, fatty liver, and acquired partial lipoatrophy suggested she was suffering from lipodystrophic syndrome.

The daily administration of recombinant methionyl human leptin (metreleptin; 0.04 mg/kg/day) was started and increased to a maintenance dose (0.08 mg/kg/day). Subsequently, all metabolic profiles, including the HbA1c and triglyceride levels, returned to the normal range.

### Case 2

A 40-year-old Japanese woman underwent partial liver transplantation from her husband as a living donor for alcoholic liver disease after abstinence from alcohol for 2 years. After transplantation, she developed non-alcoholic steatohepatitis (NASH), and the insulin therapy was initiated because of new onset of diabetes. A total daily insulin dose of 44 units was required to achieve glycemic control, and she manifested prominent insulin resistance although she was lean. NASH progressed to liver failure, and a second liver transplantation from a brain-dead donor was performed at 42 years of age. She received standard and maintenance immunosuppression regimens, including prednisolone 5–10 mg/day and tacrolimus 1–3 mg/day with trough concentration of 5–10 ng/mL.

**Fig. 3:**
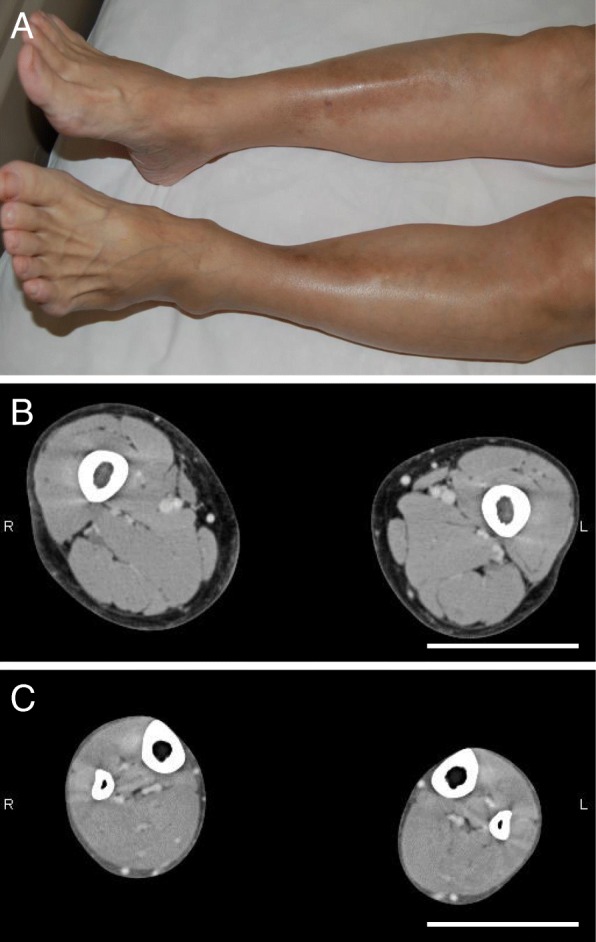
Loss of subdermal adipose tissues in the lower legs in Case 2. **a** She manifested the loss of subdermal adipose tissues of the lower legs. **b** The subdermal adipose tissues of the thighs were spared from lipoatrophy, as revealed by MRI. **c** The subdermal adipose tissues of the lower legs were almost completely lost in the lower legs, as revealed by MRI

**Fig. 4:**
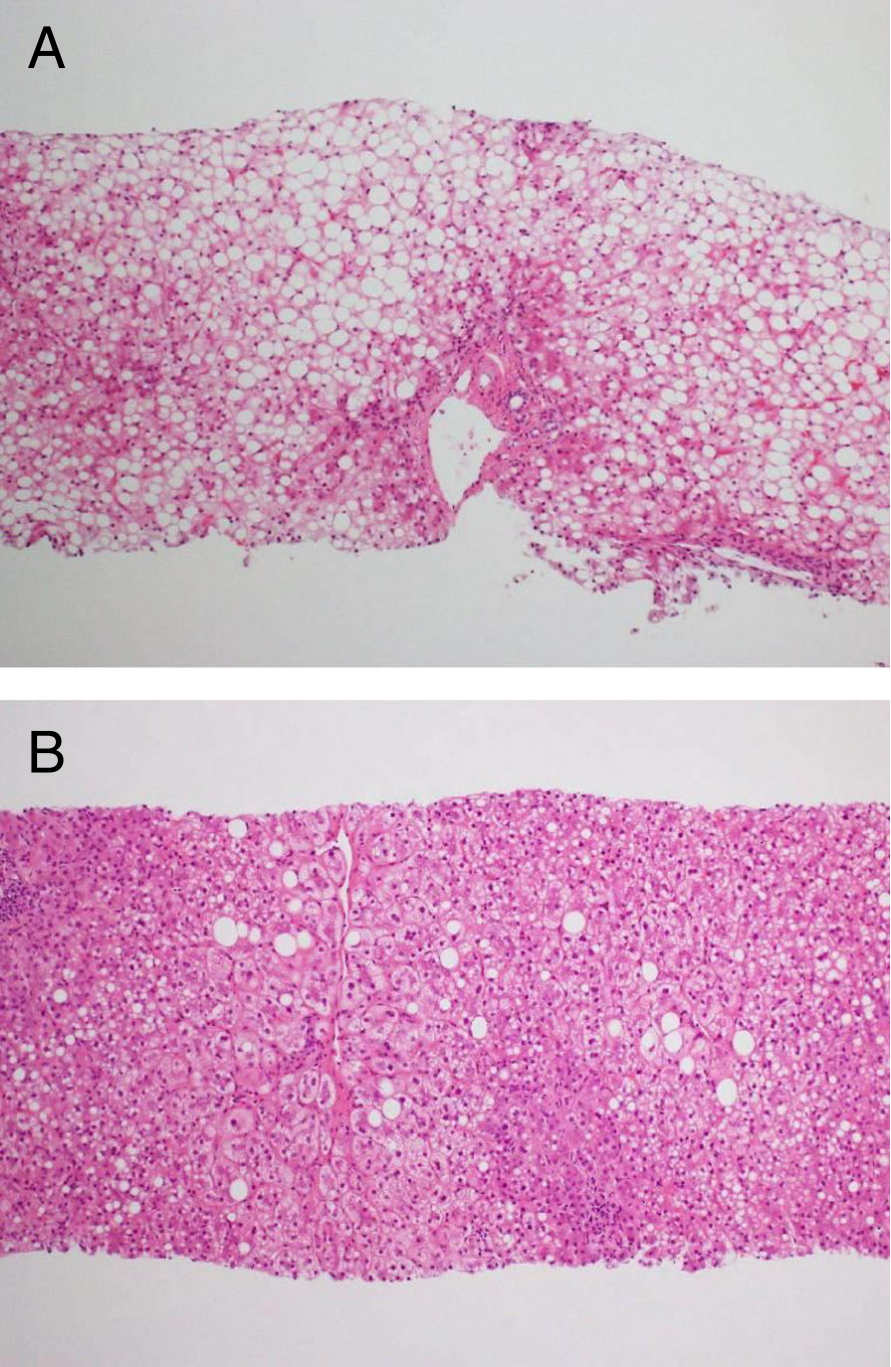
Liver biopsy samples stained with Periodic acid-Schiff stain. **a** Case 2 before treatment with metreleptin. **b** Case 2 after treatment with metreleptin. The lipid droplets in hepatocytes are significantly reduced

After her second liver transplantation, marked hypertriglyceridemia of 1000 to 1900 mg/dL developed even under combination therapy with 200 mg/day of bezafibrate and 10 mg/day of ezetimibe. She was admitted to our hospital for the treatment of NASH and hypertriglyceridemia. Under nutritional guidance, she had been on a 1600-kcal diet consisting of 60% carbohydrates, 20% protein, and 20% fat. On admission, her height was 149 cm, body weight 37.9 kg, BMI 17.1 kg/m^2^, and systemic blood pressure 143/83 mmHg. Similar to Case 1, she manifested almost complete loss of subdermal adipose tissues of the bilateral forearms and lower legs, but the upper arms, thighs, face, and trunk were spared from lipoatrophy (Fig. 3a). The almost complete loss of subdermal adipose tissues was confirmed by magnetic resonance imaging (MRI) (Fig. 3b and c). Her HbA1c was 5.3%, serum CRP 4.2 ng/ml, ΔCRP 1.9 ng/ml after the injection of glucagon, and daily urinary excretion 20.2 μg/day, while her serum leptin levels were 3.5 ng/mL (range for women, 2.5–21.8). She had elevation of liver enzymes: AST 38 IU/L, ALT 13 IU/L, and GGT 241 IU/L. She demonstrated prominent fatty liver by computed tomography and severe NASH by a liver biopsy (Fig. 2c and 4a). She also had dyslipidemia with low HDL-cholesterol (40 mg/dL) and high triglyceride (968 mg/dL), VLDL, and IDL levels (Fig. 2d). Loss of subdermal fat tissues in the extremities, NASH, and severe dyslipidemia suggested acquired partial lipoatrophy.

Metreleptin therapy (0.08 mg/kg/day) was started to prevent repeated liver failure. Her hypertriglyceridemia was ameliorated, and her fatty liver significantly improved, as demonstrated by a repeated liver biopsy (Fig. 4b).

## Discussion

The two cases were characterized by prominent insulin resistance, diabetes, hypertriglyceridemia, and severe fatty liver disease despite having a low BMI. Their partial loss of subdermal adipose tissues of the distal extremities and medical history of transplantation prompted us to give a diagnosis of acquired partial lipoatrophy.

As in the previously reported pediatric cases of acquired partial lipoatrophy after bone marrow transplantation [3–6], the clinical manifestations of the current cases suggest that acquired partial lipoatrophy associated with related metabolic disorders in adults is a symptom of GVHD. The diagnosis may be challenging, since the lipoatrophy is limited to distal extremities. Various acquired partial lipodystrophies, such as HIV protease inhibitor-associated, pressure-induced, and panniculitis, must be ruled out for a diagnosis; however, both of the present cases lacked such a medical history or associated symptoms. Furthermore, in contrast to generalized lipoatrophy, which is characterized by abnormally low leptin levels, the plasma or serum leptin levels in acquired partial lipoatrophy are low but still within the normal range, which makes the diagnosis challenging. The recently developed and transient peripheral painful neuropathy in Case 1 at 4 years after the initiation of metreleptin and the biopsy-proved autoimmune hepatitis-like lesions prior to the development of NASH in Case 2 might be indicative of other organ involvement in GVHD, suggesting that acquired lipodystrophy was a manifestation of chronic GVHD in these two cases.

A mutation in the leptin gene was found to result in massive obesity and type 2 diabetes in humans [7] as well as in rodents. The adipose tissue mass positively correlates with the serum concentration of leptin and is a marker of acute changes in the energy intake [8]. Upon increased energy intake and a subsequent increase in the adipose tissue mass, leptin is secreted by adipocytes. Once in the blood circulation, leptin crosses the blood-brain barrier and binds to leptin receptors in the hypothalamus, including supraoptic, paraventricular, periventricular, and arcuate nuclei, and lateral hypothalamus[9]. This series of events results in the inhibition of the feeding behavior and increased energy expenditure to maintain the whole-body adipose tissue mass. In addition to central activity, leptin also exerts various effects on the peripheral organs, such as the adipose tissue, muscle, liver, pancreas, cardiovascular system, and bone [10]. Primary defects in the development and differentiation of adipose tissues cause a reduction in the leptin levels, which results in prominent insulin resistance and subsequent metabolic abnormalities resembling metabolic syndrome [11].

Thus, the administration of leptin is a fundamental therapy in generalized and partial lipoatrophy patients. One currently available therapy in humans is the administration of metreleptin. The United States Food and Drug Administration (US FDA) approved the use of metreleptin to treat complications of leptin deficiency in patients with congenital or acquired generalized lipoatrophy as a replacement therapy in addition to diet therapy [12]. In contrast, the Pharmaceutical and Medical Devices Agency (PMDA) in Japan approved metreleptin to treat hyperglycemia and hypertriglycemia in patients with generalized or partial lipodystrophy [13]. The two present cases met the criteria of the PMDA and were therefore treated with metreleptin; following treatment, their metabolic abnormalities were ameliorated.

It has been suggested that this therapy may induce the development of anti-metreleptin antibodies with neutralizing activity and severe infection, which may lead to the loss of metreleptin efficacy in certain patients undergoing replacement [14]. However, both cases 1 and 2 were successfully treated with metreleptin for 4 and 3 years, respectively. Furthermore, Case 2 discontinued insulin therapy, and Case 1 was treated with 8–10 units of basal insulin.

## Conclusions

Acquired partial lipoatrophy after transplantation is a manifestation of chronic GVHD, and metreleptin is a powerful biomedicine capable of reversing the metabolic abnormalities related to prominent insulin resistance.
